# Effects of Homogenization Heat Treatment on the Fe Micro-Segregation in Ti-1023 Titanium Alloy

**DOI:** 10.3390/ma16144911

**Published:** 2023-07-09

**Authors:** Jian-Bo Tong, Chao-Jie Zhang, Jun-Shu Chen, Meng-Qi Yan, Rui-Lin Xu, Li-Jun Huang

**Affiliations:** 1 Aviation Key Laboratory of Science and Technology on Advanced Titanium Alloys, AECC Beijing Institute of Aeronautical Materials, Beijing 100095, China; 2 School of Metallurgical Engineering, Anhui University of Technology, Ma’anshan 243002, China

**Keywords:** Ti-1023 titanium alloy, homogenization heat treatment, diffusion, micro-segregation

## Abstract

The segregation of the Fe element in Ti-10V-2Fe-3Al titanium alloy (Ti-1023) can lead to the generation of beta flecks, which seriously affects the performance of Ti-1023 products. During the heat treatment (HT) process at a high temperature, the Fe element in Ti-1023 ingots will migrate, making its distribution more uniform and reducing the segregation index. In this paper, the control of Fe micro-segregation in Ti-1023 ingots by homogenization HT was investigated. Firstly, dissection sampling and SEM-EDS analysis methods were used to study the distribution pattern of the Fe element in the equiaxed grains in the core of Ti-1023 ingots. It was found that the Fe content in the grain gradually increased along with the radial direction from the core to the grain boundary. Then, the homogenization HT experiments and numerical simulations of Ti-1023 at different HT temperatures from 1050 °C to 1200 °C were carried out. The results showed that the uniformity of Fe element distribution within grain can be significantly improved by the homogenization HT. With increasing HT temperature, Fe atoms migration ability increases, and the uniformity of Fe element distribution improves. Homogenization HT at 1150 °C and 1200 °C for 12 h can effectively reduce the degree of Fe element segregation.

## 1. Introduction

Ti-1023 is a typical near-beta Ti alloy with a beta transit temperature of 795 °C and has high fracture toughness, deep hardening potential and inherent ductility [1,2,3]. It is mainly used in the manufacturing of large load-bearing components such as aircraft fuselages, helicopter rotors and landing gears [4,5,6,7,8]. Due to the small equilibrium distribution constant of Fe during solidification of Ti-1023, which is as low as 0.3, it has a high tendency of segregation [9,10,11,12]. The low partition ratios of Fe, combined with the relative movement of liquid and solid phases in the two-phase zone caused by buoyancy and Lorentz force, large differences of Fe content at different areas and β-flecks are likely to occur in the ingot [13,14,15,16,17] result in the precipitation of brittle phase. The β-flecks usually deteriorate the plasticity and fatigue properties of Ti-1023 forgings [14].

At the microscopic scale, there is a possibility of relatively significant compositional differences within individual grains due to different solidification sequences, which could be inherited in bars and forgings [18,19,20,21,22]. It has been found that there is a certain degree of segregation of Fe elements in Ti-1023. Zhao Yongqing et al. [23] studied the distribution of Fe elements in Ti-3Fe and Ti-6Al-1.7Fe within isometric crystals, and the results proved that the two alloys have a high Fe element content in the ingot near the grain boundary. For the Ti-3Fe alloy with a high Fe element content, the elevated Fe element content near the grain boundary was more obvious. Jing Zhenquan et al. [24] examined the heredity law of macrosegregation of the easily segregated Fe element between the primary and secondary ingots by numerical simulation of the interaction between temperature and solute fields during the process of vacuum arc remelting.

Multiple smelting, homogenization HT and other methods are required to prevent and control the segregation of Fe in Ti-1023 [25,26,27,28]. HT can relieve macro-segregation and micro-segregation of many kinds of alloys to a certain degree [29,30,31,32,33]. However, at present, there are limited reports on the intragranular distribution of Fe in Ti-1023, and there is little research on the improvement of intragranular segregation by annealing. It is important to reveal the distribution pattern of Fe content in the grains of Ti-1023 ingots to study the formation and control of β-flecks. Therefore, this paper uses energy spectroscopy to study the distribution pattern of Fe content at the grain scale in large-size Ti-1023 ingots. Various homogenization HT processes were used to eliminate or reduce the gradient of Fe content within the grains.

## 2. Materials and Methods

### 2.1. Sampling and Corrosion of Ti-1023 Ingots

Ti-1023, with the composition shown in Table 1, was smelted using a vacuum smelting furnace and cast into an ingot with the size of Ф360 mm × 1300 mm. A pie-shaped sample with the size of Ф360 mm × 10 mm was cut from the middle part of the Ti-1023 ingot.

**Figure 1:**
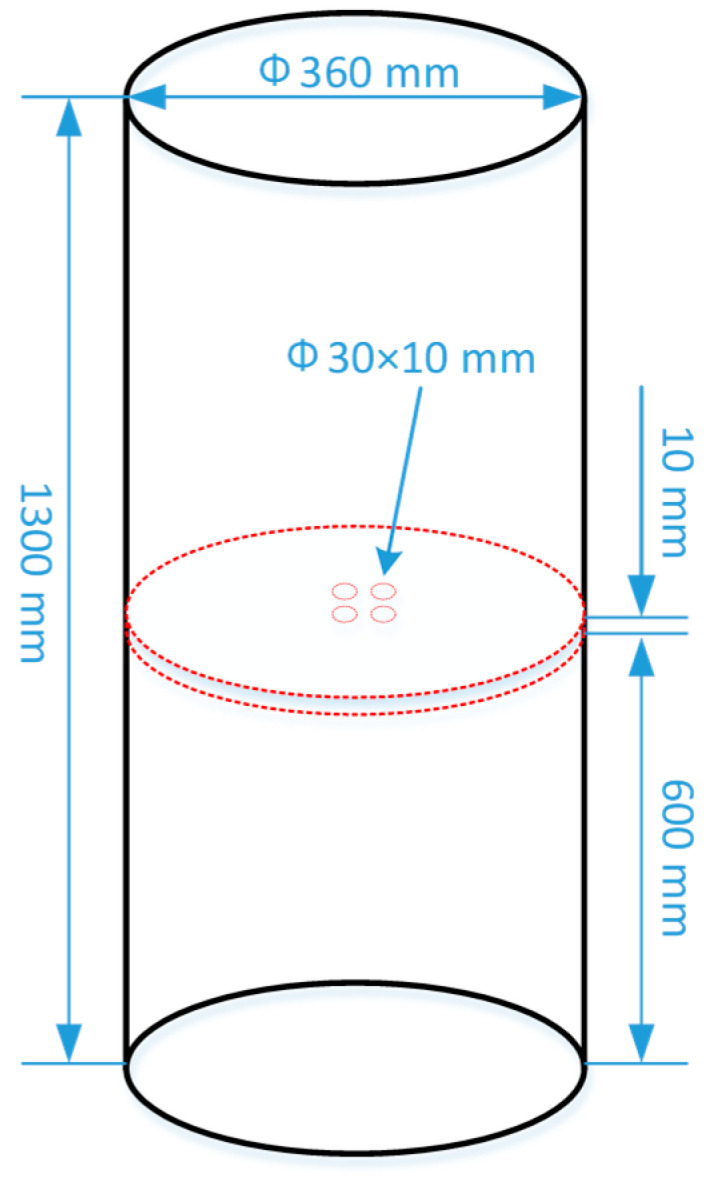
Schematic of sampling from the Ti-1023 ingot.

Considering the segregation in the axial part of the ingot is obvious [17], four cylindrical samples with a size of Ф30 mm × 10 mm were cut from the center of the pie-shaped sample, as shown in Figure 1.

In order to observe the grain distribution of the Ti-1023 sample, the sample was ground using silicon carbide abrasive paper, polished using the diamond polishing paste, and finally etched using 4% nitric acid alcohol solution.

### 2.2. Detection of Fe Element Content Distribution in Sample Grains

**Figure 2:**
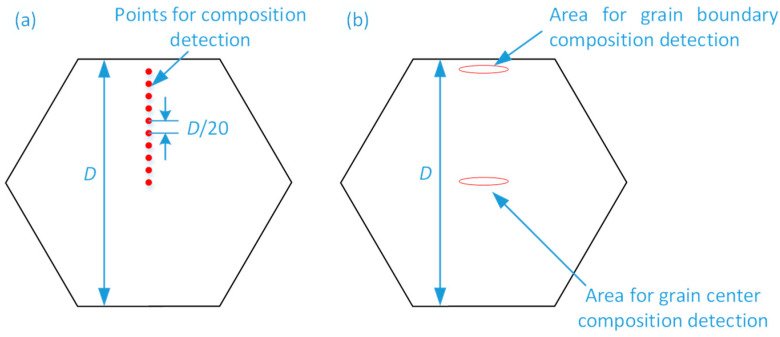
Schematic of composition detection areas (**a**) Micro-areas chosen in one grain for composition detection, (**b**) Composition detection areas in grain center and near grain boundary.

The microstructure of the four samples mentioned above were observed using SEM (JSM-6510LV). In each sample, three grains with a regular shape were chosen to detect the Fe element content. The distances between the parallel edges of the grains were measure as *D*, and then several micro-areas with the size of 10 μm × 10 μm were selected from the midpoint of one of the parallel edges along the direction of the grain center at intervals of *D*/20, as shown in Figure 2a. The compositions of each micro-area were detected using an energy spectrum analyzer (JSM-6510LV, manufacturer is Hitachi, Tokyo, Japan).

### 2.3. Homogenization HT of Sample

Before the homogenization HT, select three grains with large size about 6 mm to 10 mm in each sample. For each grain, the composition was detected using an energy spectrum analyzer at the grain center and near grain boundary, as shown in Figure 2b.

The four samples, numbered 1# to 4#, were encapsulated in different quartz tubes filled with argon as a protective atmosphere. Keep the four quartz tubes at different temperatures of 1050 °C, 1100 °C, 1150 °C and 1200 °C for 12 h, and then cooled to room temperature in the furnace.

After the homogenization HT, the four samples were taken out of the quartz tubes. In the same way, the samples were etched using Carroll solution after being ground and polished. Three grains with a large size about 6 mm to 10 mm were chosen in each sample, and their compositions were detected using an energy spectrum analyzer at the grain center and near the grain boundary, as shown in Figure 2b.

## 3. Numerical Simulation of Fe Diffusion Intragranular of Ti-1023 during Homogenization HT

In order to establish a mathematical model for the diffusion and transport of Fe in Ti-1023, the following assumptions were made:

1. Grain boundary and dislocation have no influences on diffusion.
2. The diffusion rate of Fe is equal in all directions.
3. The diffusion coefficient of Fe is a constant value when the temperature and composition are determined.

Based on Fick’s law, the mathematical model of Fe diffusion in Ti alloy was established, and the main governing equation is the component transfer equation, as shown in Formula (1):

(1) $$\frac{\partial C}{\partial t}=\frac{\partial }{\partial x_{i}}\left(D\frac{\partial C}{\partial x_{i}}\right)$$

where *C* is the concentration of Fe, mol·m^−3^, *x* is the coordinate of *i* direction, m, *D* is the diffusion coefficient, m^2^·s^−1^, which can be calculated via Formula (2):

(2) $$D=D_{0}\exp\left(-\frac{Q}{RT}\right)$$

**Figure 3:**
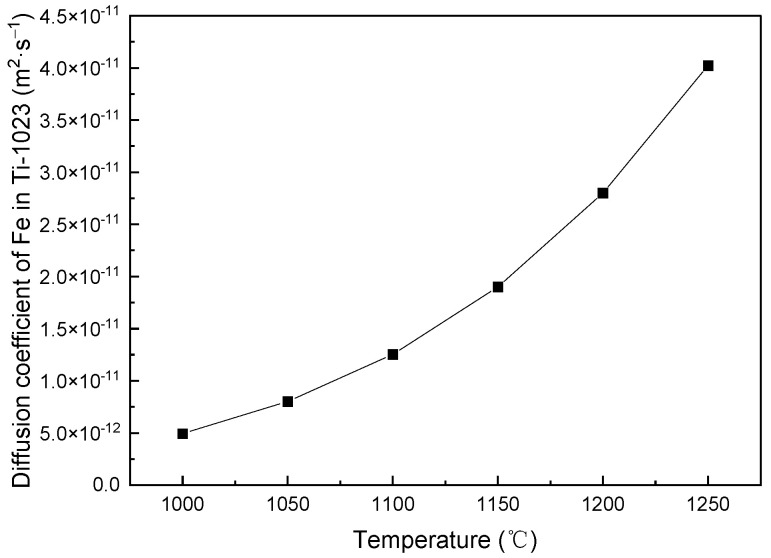
Diffusion coefficients of Fe element in Ti-1023 during homogenization HT.

The diffusion constant *D*_0_ and the activation energy of the Fe element in Ti-1023 are obtained through diffusion couple test, which are 5.45 × 10^−5^ m^2^·s^−1^ and 249,940 J·mol^−1^, respectively. Figure 3 shows the diffusion coefficients of the Fe element in Ti-1023 at different temperatures during the homogenization HT.

**Figure 4:**
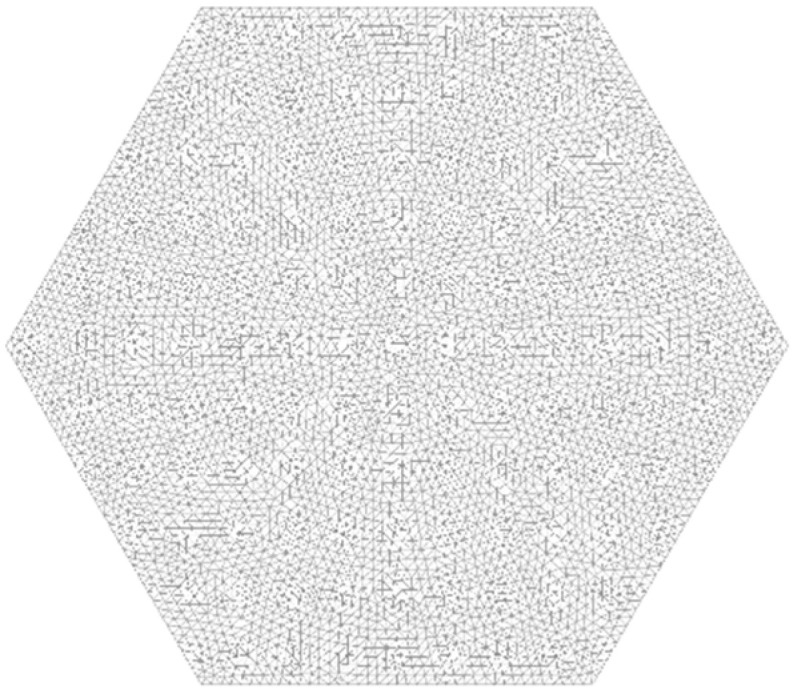
Geometric model and mesh of a single grain of Ti-1023.

The mathematical forms of the unsteady diffusion mass transfer and unsteady heat conduction equations are similar. Therefore, the heat transfer module in the finite element software ProCAST can be used for the numerical simulation of Fe diffusion during the Ti-1023 homogenization HT process. A two-dimensional geometric model of a single grain of Ti-1023 was established, assuming the grain to be hexagonal with a side length of 4 mm. The finite element mesh of the grain consisted of 10,704 nodes and 9142 triangular elements, which were selected based on several mesh refinements, as shown in Figure 4.

**Figure 5:**
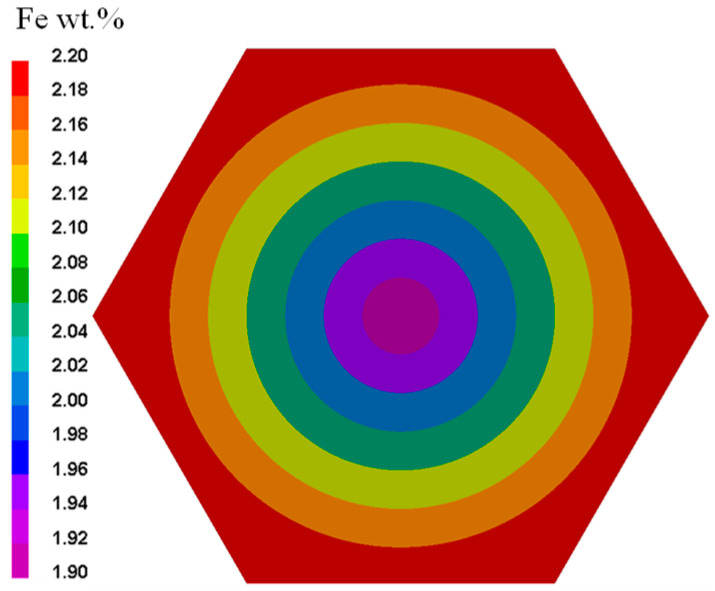
Initial distribution of Fe element content.

The initial distribution of Fe contents is shown in Figure 5. The Fe contents in the grain center and grain boundary are set according to the results of detection of Fe element content distribution in sample grains, which are about 1.9% and 2.2%, respectively. The boundary edges of the single grain are considered walls, meaning that no Fe atoms moving in or out of the single grain through these six edges.

## 4. Results and Discussion

### 4.1. Characterization of Intragranular Fe Content Distribution of Ti-1023 Ingot

**Figure 6:**
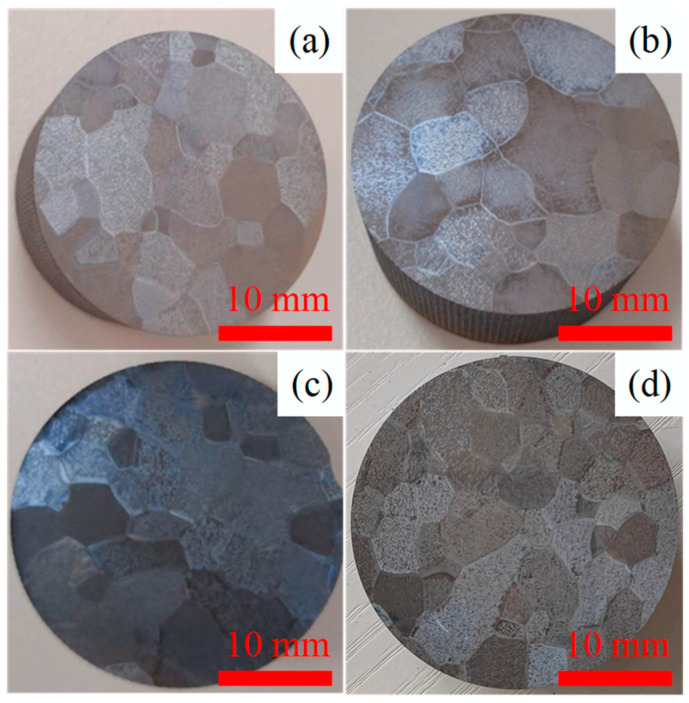
Macrographs of grain distributions of the four samples from the Ti-1023 ingot, (**a**–**d**) are 1# sample to 4# sample, respectively.

Macrographs of the grain distributions of the four samples from the Ti-1023 ingot are shown in Figure 6. It can be seen that the shape of the grains is irregular polygon, and the measured sizes of the grains using area method are ranging from about 3 mm to 8 mm.

**Figure 7:**
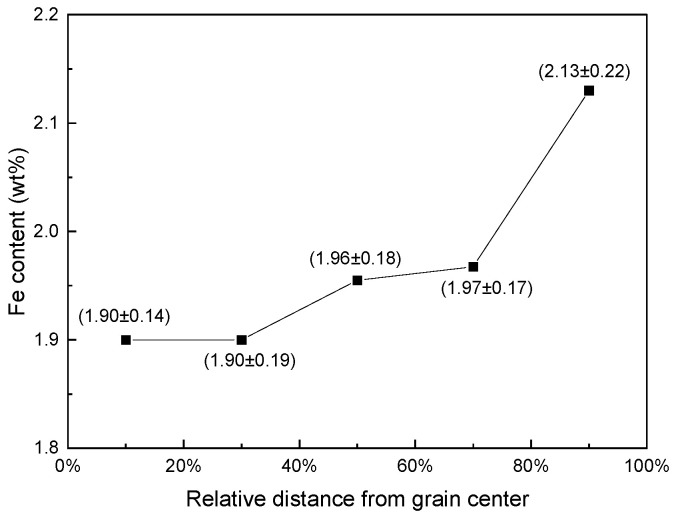
Average distribution of Fe content in the grains along the radial direction.

The distributions of Fe content in the grains of the Ti-1023 samples are shown in Table 2. It can be seen that the distribution of Fe content in the grains is uneven, and in most of the grains, the Fe content is low in the grain center and high near the grain boundary. The average distribution of Fe content in the grains along the radial direction is obtained by averaging the Fe content at the corresponding position of all grains, as shown in Figure 7. It can be seen that the Fe content in the grain increases gradually from the center to the boundary along the radial direction. Put differently, there is positive segregation of Fe content near the grain boundary and negative segregation near the grain center. The average Fe content increases from 1.90% near the center to 2.15% near the grain boundary.

The segregation of the Fe element in Ti-1023 is caused by the small equilibrium distribution constant *K*_0_ of the Fe element during solidification. *K*_0_ is the ratio of Fe content in the solid and liquid phases at equilibrium, that is, *K*_0_ = *C*_S_/*C*_L_, where *C*_S_ and *C*_L_ are the concentration of solute in the solid and liquid phases, respectively. The difference in Fe content between the grain center and the grain boundary has an important influence on the formation of β-freckle, which would appear during the subsequent forging process.

### 4.2. Effect of Homogenization HT on the Distribution of Fe Content in the Grains

The Fe contents in the grain center and boundary of the Ti-1023 ingot before and after homogenization HT were measured, and the Fe content distributions in the grain center and near the grain boundary of 1# to 4# samples before and after HT were obtained, as shown in Table 3. The Fe content deviation is used to characterize the difference in Fe content between at the grain center and grain boundary, which is calculated with the formula (*C*_boundary_ – *C*_center_)/*C*_boundary_ × 100%, where *C*_boundary_ and *C*_center_ represent the Fe contents in grain boundary and grain center, respectively. For the samples before HT, according to the statistical results of 36 pairs of Fe contents in the grain boundary and in the center of 12 grains, it can be seen that there is a difference of 3.8–13.3% between the Fe content in the grain center and the grain boundary. In other words, the Fe element exhibits positive segregation at the grain boundary of the Ti-1023 ingot.

**Figure 8:**
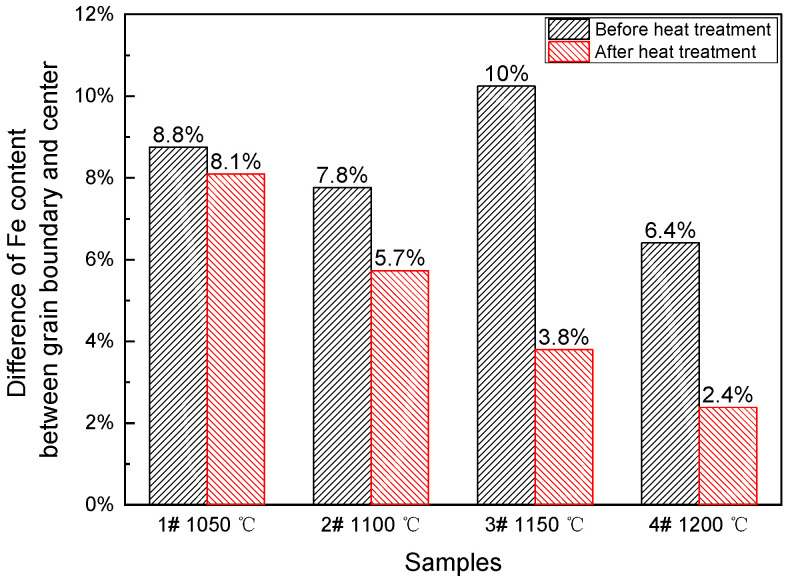
Fe content deviations before and after homogenization HT.

Then, the 1#–4# Ti-1023 samples were subjected to homogenization HT at different temperatures of 1050 °C, 1100 °C, 1150 °C and 1200 °C for 12 h, respectively. During the homogenization HT process, Fe atoms are capable of diffusing against the concentration gradient, reducing the chemical segregation to a certain extent [34,35]. The results show that after homogenization HT at 1050 °C for 12 h, the Fe content deviations in the Ti-1023 ingot are 5.7%, 12.8% and 5.6%, respectively, which are not significantly lower than before HT. These results indicate that at 1050 °C, the diffusivity of the Fe element is lower, and the diffusion rate is lower, resulting in an insignificant homogenization effect of the Fe content after 12 h. When the homogenization HT temperature is increased to 1100 °C, the Fe content deviations in the ingot are 3.4%, 4.6% and 9.3%, respectively, which are slightly improved compared to before HT. This indicates that significant homogenization and diffusion of the Fe element cannot be achieved within 12 h under 1100 °C. When the homogenization HT temperature is increased to 1150 °C, the Fe content deviations in the ingot are 2.6%, 3.8% and 5.0%, respectively. The uniformity of the Fe content distribution in the grains is improved to a certain extent compared to before HT. After 12 h of homogenization HT at 1200 °C, the Fe content deviations in the ingot reaches 0.4–3.8%, and the distribution uniformity of the Fe content is further improved with the increase in temperature, as shown in Figure 8.

According to the above analysis of the experimental data, the Fe content deviations of the Ti-1023 samples after HT decrease gradually with the increase in HT temperature. The homogenization HT above 1150 °C for 12 h can make more sufficient Fe diffusion. With the increase in HT temperature, the atomic diffusion energy is gradually activated, and atomic mobility is gradually improved [36,37,38]. The diffusion coefficient of the Fe element in the Ti alloy matrix increases exponentially with the increase in temperature, thus increasing the HT temperature can improve the diffusion of the Fe element effectively and improves the uniformity of its distribution.

However, in actual industrial production of Ti-1023, the oxidation of the ingot during the HT process would cause the loss of the metal and reduce the yield of the Ti alloy. Therefore, the homogenization HT temperature should be determined according to the degree of oxidation and the effect of the Fe content deviation.

### 4.3. Homogenization Factors of Fe Content in Ti-1023 Grains during HT

#### 4.3.1. Effect of HT Temperature on Homogenization of Fe in Grains

**Figure 9:**
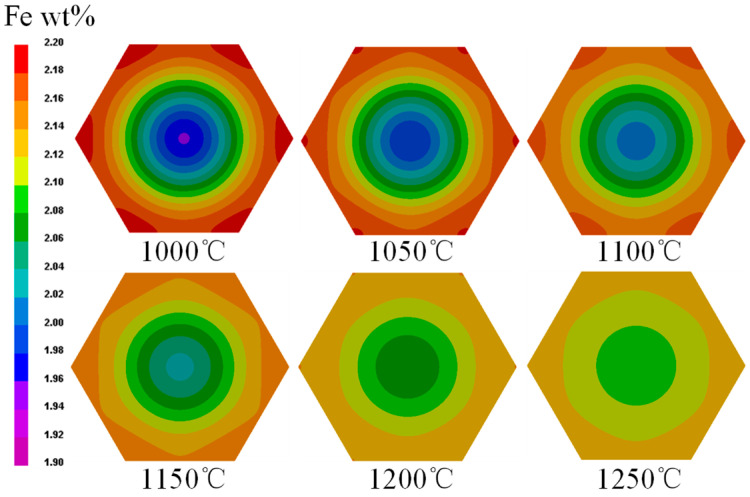
Distributions of Fe content in Ti-1023 grains after homogenization HT for 12 h at different temperatures.

Both HT temperature and time have significant effect on the homogenization of alloys [39,40]. Increased homogenization treatment temperature and extended holding time can significantly alleviate the segregation of the alloying element [41]. In other words, a reasonable high HT temperature and long HT time will result in a uniform distribution of alloy elements. The distributions of Fe content in Ti-1023 grains after homogenization HT for 12 h at different temperatures are shown in Figure 9, it can be seen that: (1) For a single grain, the higher the homogenization HT temperature, the more uniform the distribution of Fe element content would be after a certain HT time. (2) Homogenization HT temperature has a great influence on the homogenization of Fe content in a single grain. For example, after homogenization HT at 1000 °C and 1250 °C for 12 h, the differences in Fe contents between the grain center and grain boundary are 0.21% and 0.04%, respectively, and the Fe content deviations are 10.9% and 1.8%, respectively.

**Figure 10:**
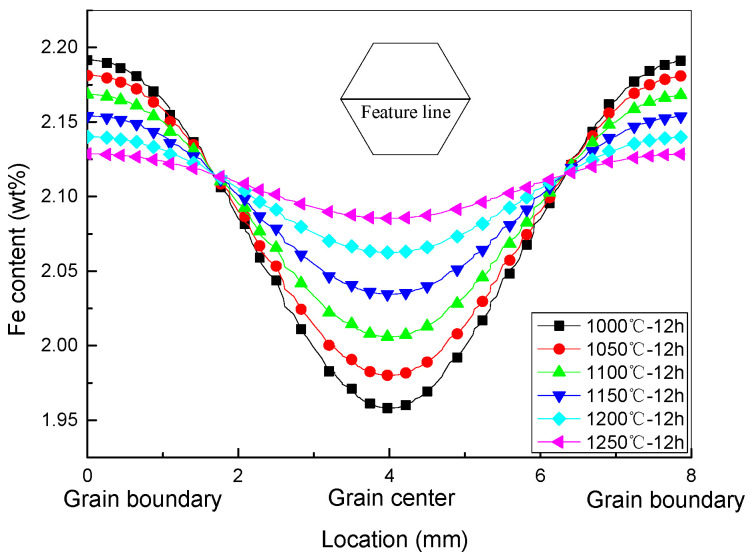
Fe content distribution curve along the characteristic line after homogenization HT at different temperatures, 12 h.

In order to further clarify the effect of homogenization HT temperature on the distribution of Fe content within the grains of Ti-1023, a characteristic line passing through the core of the grain was selected, and the Fe content distribution curve along this characteristic line after homogenization HT at different temperatures are plotted, as shown in Figure 10. It can be seen that when the homogenization HT time is fixed, the higher the temperature, the more uniform the Fe content distribution along the characteristic line. For example, when the homogenization HT time is 12 h, the extreme difference in Fe content along the characteristic line at 1000 °C is 0.19%, while at 1250 °C is 0.01%.

**Figure 11:**
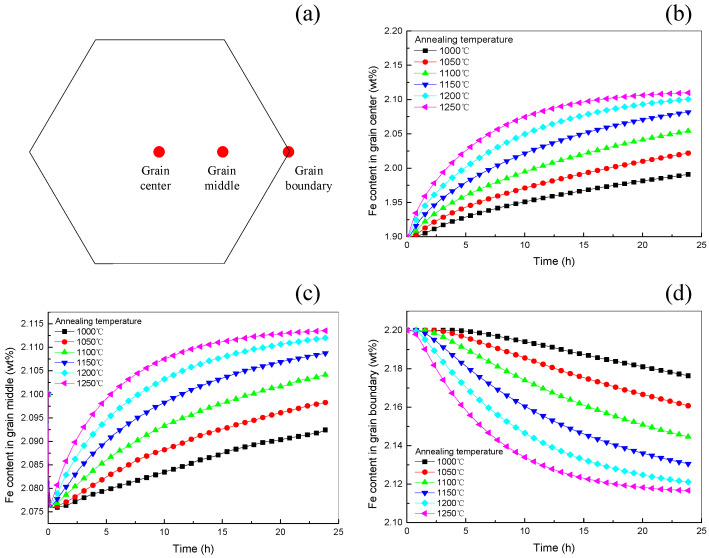
Fe content of characteristic points at different homogenization HT temperatures, (**a**) Schematic of three characteristic points selected within the grain of Ti-1023, (**b**–**d**) Fe contents at different homogenization HT temperatures with time of the core, the middle, and the grain boundary, respectively.

Three characteristic points were selected within the grain of Ti-1023: the core, the middle and the grain boundary, as shown in Figure 11a. The Fe contents of these three characteristic points at different homogenization HT temperatures with time are shown in Figure 11b–d. It can be seen that: (1) The Fe content in the core and middle of the grain increases with increasing homogenization HT time. The higher the homogenization HT temperature, the faster the initial increase in Fe element content, and the slower the subsequent increase at the end of the HT. (2) The Fe content at the grain boundaries decreases with increasing homogenization HT time. During homogenization HT, the high temperature accelerates the diffusion rate of atoms, making the dendritic segregation gradually disappear [42]. The higher the homogenization HT temperature, the faster the initial decrease in Fe content, and the slower the subsequent decrease at the end of the HT. 

#### 4.3.2. Effect of HT Time on Homogenization of Fe in Grain

**Figure 12:**
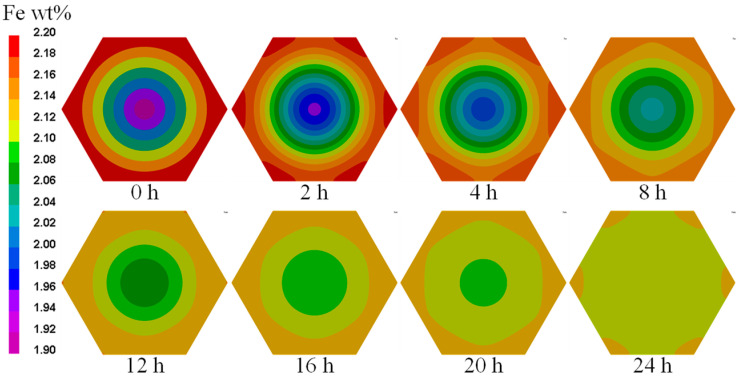
Distributions of Fe content in grain during homogenization HT of Ti-1023 at the temperature of 1200 °C.

At a reasonable homogenization temperature, the alloy elements gradually tend to distribute uniformly with an increase in homogenization time [42]. Distributions of Fe content in the grain during homogenization HT of Ti-1023 were obtained at different temperatures of 1000 °C, 1050 °C, 1100 °C, 1150 °C, 1200 °C and 1250 °C, respectively. Figure 12 shows distributions of Fe content in the grain during homogenization HT of Ti-1023 at a temperature of 1200 °C. During homogenization HT of Ti-1023, the Fe content in the grain tends to become more uniform gradually with an increase in time. When the HT time is 24 h at 1200 °C, the Fe content in the Ti-1023 grains can reach a nearly complete uniformity.

**Figure 13:**
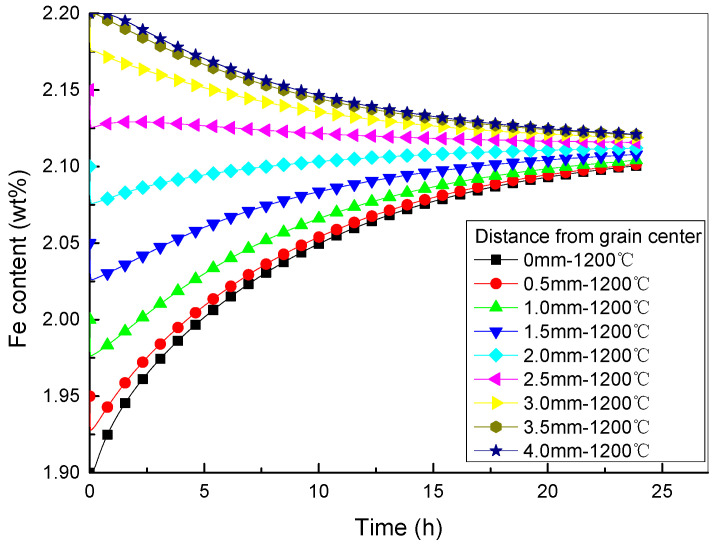
Fe content distributions at different positions in the grain during homogenization HT at 1200 °C.

Fe content distributions at different positions in the grain during homogenization HT of Ti-1023 are shown in Figure 13. It can be seen that: (1) At a certain HT temperature, the Fe content at different distances from the grain center approaches the average value over time. (2) When the HT temperature is high, such as 1200 °C, the Fe content at different distances from the grain center exhibits a nonlinear change with time, following a concave curve.

## 5. Conclusions

In this paper, the distribution of Fe content in the grains of Ti-1023 ingots smelted by vacuum self-consumption was investigated. The distribution pattern of Fe contents along the radial direction of the grains in the Ti-1023 ingot was obtained. The effect of homogenization HT temperature on reducing Fe segregation in the microscopic region was investigated by conducting homogenizing HT at temperatures ranging from 1050 °C to 1200 °C for 12 h. Combined with the numerical simulation, the Fe diffusion during the homogenization HT of Ti-1023 was investigated to explore the Fe diffusion law within the grain. The influences of the homogenization HT temperature and time on the distribution of Fe content in Ti-1023 were clarified and the following conclusions were drawn:

1. The Fe content in the Ti-1023 ingots near the grain boundary is higher than that in the core of the grain.
2. The degree of uniform distribution of Fe content within the grain can be improved by homogenization HT. As the HT temperature increases, the Fe element migration capacity improves and the degree of uniform distribution of Fe elements increases.
3. Homogenization HT time has a great influence on the distribution of Fe content within the grain. The longer the HT time, the more uniform the distribution of Fe content. Fe content can achieve complete uniformity within the grain of Ti-1023 after homogenization HT at 1200 °C for 12 h.
4. Homogenization HT of Ti-1023 at 1150 °C to 1200 °C for 12 h can reduce the Fe content deviation from about 10% to less than 4%.

In order to save heat treatment costs and maximize material performance, further research will be conducted on the plasticity and fatigue properties of Ti-1023 with different degrees of Fe segregation after homogenization HT. In addition, methods for controlling Fe segregation during the solidification process of Ti-1023 ingots will also be investigated in future studies.

## Figures and Tables

**Table 1:** Composition of Ti-1023, wt.%.

Element	Al	V	Fe	O	N	H	Ti
Content	3.02 ± 0.03	10.25 ± 0.05	1.92 ± 0.10	0.08 ± 0.01	0.009 ± 0.001	0.0012 ± 0.0003	Bal.

Note: The Al and V contents were measured using the method of inductively coupled plasma atomic emission spectroscopy. The Fe content was measured using the method of atomic absorption spectroscopy. The O, N and H contents were measured using the methods of inert gas melting and infrared detection.

**Table 2:** Distributions of Fe content in grains of the Ti-1023 samples, wt%.

Relative Distance from Grain Center	Sample1#-Grain1#	Sample1#-Grain2#	Sample2#-Grain1#	Sample2#-Grain2#	Sample3#-Grain1#	Sample3#-Grain2#	Sample4#-Grain1#	Sample4#-Grain2#
0%	1.81	2.15	1.95	1.69	1.93	1.92	2.20	2.10
10%	2.04	1.94	2.15	1.78	1.67	2.02	1.80	1.80
20%	1.97	1.91	2.12	1.58	1.83	1.84	1.90	1.80
30%	1.84	1.89	2.16	1.87	1.69	1.55	2.00	2.20
40%	1.89	1.89	2.22	1.84	1.80	1.71	2.10	2.00
50%	1.76	2.28	2.10	1.82	1.65	2.13	1.90	2.00
60%	1.66	1.97	2.13	1.86	1.96	1.79	1.80	1.80
70%	1.77	2.29	2.11	1.92	1.68	1.87	2.00	2.10
80%	2.10	2.48	2.07	1.68	1.68	1.92	1.70	1.60
90%	1.98	2.49	2.23	1.81	2.04	1.99	2.50	2.00
100%	1.90	2.28	2.25	1.59	1.99	2.13	2.50	1.50

**Table 3:** Fe content in the grain center and boundary before and after homogenization HT.

Sample#	Before HT			After HT			HT Temperature, °C
	Grain Boundary	Grain Center	Difference	Grain Boundary	Grain Center	Difference	
1#	2.16	1.96	9.3%	2.10	1.98	5.7%	1050
1#	2.22	2.04	8.1%	2.18	1.90	12.8%	1050
1#	2.36	2.15	8.9%	2.14	2.02	5.6%	1050
2#	2.20	1.96	10.9%	2.08	2.01	3.4%	1100
2#	2.17	2.01	7.4%	2.40	2.29	4.6%	1100
2#	2.20	2.09	5.0%	2.15	1.95	9.3%	1100
3#	2.12	2.01	5.2%	2.27	2.21	2.6%	1150
3#	2.17	1.91	12.0%	2.38	2.29	3.8%	1150
3#	2.25	1.95	13.3%	2.20	2.09	5.0%	1150
4#	2.24	2.02	9.8%	2.11	2.03	3.8%	1200
4#	2.38	2.29	3.8%	2.14	2.07	3.3%	1200
4#	2.40	2.26	5.8%	2.46	2.45	0.4%	1200

## Data Availability

Not applicable.

## References

[1] Neelakantan S., San Martin D., Rivera-Diaz-del-Castillo P., van der Zwaag S. (2009). Plasticity Induced Transformation in a Metastable Beta Ti-1023 Alloy by Controlled Heat Treatments. Mater. Sci. Technol..

[2] Wang Q., Yang C., Wu J., Gao B., He Z., Wang K. (2022). Phase Transformation Behavior of Ti-1023 Under Static Heat Treatment and Dynamic Thermo-Mechanical Coupling. Mater. Charact..

[3] Yang H., Chen Z., Zhou Z. (2015). Influence of Cutting Speed and Tool Wear On the Surface Integrity of the Titanium Alloy Ti-1023 During Milling. Int. J. Adv. Manuf. Technol..

[4] Lhadi S., Purushottam raj purohit R.r.p., Richeton T., Gey N., Berbenni S., Perroud O., Germain L. (2020). Elasto-Viscoplastic Tensile Behavior of as-Forged Ti-1023 Alloy: Experiments and Micromechanical Modeling. Mater. Sci. Eng. A.

[5] Li L., Wang Z., Ma W. (2022). Experimental Study on the High Temperature Impact Torsional Behavior of Ti-1023 Alloy. Materials.

[6] Li P., Sun X., Zhang T., Zhang H., Wang D., Sun Q., Xiao L., Sun J. (2019). Adaptive Volume Control in Titanium Alloy for High Temperature Performance. Materials.

[7] Quan F., Chen Z., Zhu Y., Zhang Y. (2019). A Method of Assessing the Strength of Metal Surface Using Film Samples On Titanium Alloy Ti-1023. Strain.

[8] Storchak M., Zakiev I., Träris L. (2018). Mechanical Properties of Subsurface Layers in the Machining of the Titanium Alloy Ti10V2Fe3Al. J. Mech. Sci. Technol..

[9] Bermingham M.J., McDonald S.D., StJohn D.H., Dargusch M.S. (2009). Segregation and Grain Refinement in Cast Titanium Alloys. J. Mater. Res..

[10] Joseph S., Kontis P., Chang Y., Shi Y., Raabe D., Gault B., Dye D. (2022). A Cracking Oxygen Story: A New View of Stress Corrosion Cracking in Titanium Alloys. Acta Mater..

[11] Ng C.H., Bermingham M.J., Dargusch M.S. (2021). Eliminating Segregation Defects During Additive Manufacturing of High Strength Beta-Titanium Alloys. Addit. Manuf..

[12] Ng C.H., Bermingham M.J., Yuan L., Dargusch M.S. (2022). Towards Beta-Fleck Defect Free Additively Manufactured Titanium Alloys by Promoting the Columnar to Equiaxed Transition and Grain Refinement. Acta Mater..

[13] Fan K., Wu L.C., Li J.J., Wang J.C. (2020). Numerical Simulation of Macrosegregation Caused by Buoyancy Driven Flow During Var Process for Titanium Alloys. Rare Metal Mat. Eng..

[14] He Y., Hu R., Luo W., Sun F., Wang K., Fu B., Li J., Liu X. (2017). Effect of Stirring Magnetic Field On the Macrostructure and Macrosegregation of Fe Element of Ti-1023 Alloy Ingot. Rare Metal Mat. Eng..

[15] Liao Q., Ge P., Liu Y., Wang R.Q. (2020). Fe Elemental Segregation Control of Large Sized Ti70 Alloy Ingot. Rare Metal Mat. Eng..

[16] Liu X., Feng G., Zhou Y., Fan Q. (2019). Macrosegregation and the Underlying Mechanism in Ti-6.5Al-1.0Cr-0.5Fe-6.0Mo-3.0Sn-4.0Zr Alloy. Prog. Nat. Sci..

[17] Yang Z., Kou H., Li J., Hu R., Chang H., Zhou L. (2011). Macrosegregation Behavior of Ti-10V-2Fe-3Al Alloy During Vacuum Consumable Arc Remelting Process. J. Mater. Eng. Perform..

[18] Gao J.H., Nutter J., Liu X.G., Guan D.K., Huang Y.H., Dye D., Rainforth W.M. (2018). Segregation Mediated Heterogeneous Structure in a Metastable Beta Titanium Alloy with a Superior Combination of Strength and Ductility. Sci. Rep..

[19] Huang S.S., Ma Y.J., Zhang S.L., Qi M., Lei J.F., Zong Y.P., Yang R. (2019). Influence of Alloying Elements Partitioning Behaviors On the Microstructure and Mechanical Properties in Alpha Plus Beta Titanium Alloy. Acta Metall. Sin..

[20] Prithiv T.S., Kloenne Z., Li D.A., Shi R.P., Zheng Y.F., Fraser H.L., Gault B., Antonov S. (2022). Grain Boundary Segregation and its Implications Regarding the Formation of the Grain Boundary Alpha Phase in the Metastable β-Titanium Ti-5Al-5Mo-5V-3Cr Alloy. Scr. Mater..

[21] Wang J., Qin Z.W., Xiong F.H., Wang S.S., Lu X.G., Li C.H. (2018). Design and Preparation of Low-Cost Alpha Plus Beta Titanium Alloy Based On Assessment of Ti-Al-Fe-Cr System. Mater. Sci. Eng. A.

[22] Xu Z., Wang H., Tang H., Cheng X., Zhu Y. (2022). Microstructure, Microsegregation and Mechanical Properties of Directed Energy Deposited Ti-32Mo Titanium Alloy. J. Mater. Sci..

[23] Zhao Y., Liu J., Zhou L. (2005). Analysis On the Segregation of Typical Alloying Elements of Cu, Fe and Cr in Ti Alloys. Rare Metal Mat. Eng..

[24] Jing Z., Sun Y., Song S. (2023). Numerical Simulation of Macrosegregation Heredity On Tc4 Titanium Alloy Ingot by Vacuum Arc Remelting. Metall. Res. Technol..

[25] Edalati K., Daio T., Lee S., Horita Z., Nishizaki T., Akune T., Nojima T., Sasaki T. (2014). High Strength and Superconductivity in Nanostructured Niobium–Titanium Alloy by High-Pressure Torsion and Annealing: Significance of Elemental Decomposition and Supersaturation. Acta Mater..

[26] Lei Z., Chen Y., Ma S., Zhou H., Liu J., Wang X. (2020). Influence of Aging Heat Treatment On Microstructure and Tensile Properties of Laser Oscillating Welded Tb8 Titanium Alloy Joints. Mater. Sci. Eng. A.

[27] Mitchell A. (2012). Composition Control in Titanium Alloys. Proceedings of the Ti-2011: 12th World Conference on Titanium.

[28] Schwaighofer E., Schloffer M., Schmoelzer T., Mayer S., Lindemann J., Guether V., Klose J., Clemens H. (2012). Influence of Heat Treatments On the Microstructure of a Multi-Phase Titanium Aluminide Alloy. Pract. Metallogr..

[29] Dichtl C., Zhang Z.B., Gardner H., Bagot P., Radecka A., Dye D., Thomas M., Sandala R., Da Fonseca J.Q., Preuss M. (2020). Element Segregation and Alpha_2_ Formation in Primary Alpha of a Near-Alpha Ti-Alloy. Mater. Charact..

[30] Karthik G.M., Kim H.S. (2021). Heterogeneous Aspects of Additive Manufactured Metallic Parts: A Review. Met. Mater. Int..

[31] Lukyanov A.V., Pushin V.G., Kuranova N.N., Svirid A.E., Uksusnikov A.N., Ustyugov Y.M., Gunderov D.V. (2018). Effect of the Thermomechanical Treatment On Structural and Phase Transformations in Cu–_14_Al–_3_Ni Shape Memory Alloy Subjected to High-Pressure Torsion. Phys. Met. Metallogr..

[32] Xu M., Liu G.-H., Li T.-R., Wang B.-X., Wang Z.-D. (2019). Microstructure Characteristics of Ti–43Al Alloy During Twin-Roll Strip Casting and Heat Treatment. Trans. Nonferrous Met. Soc. China.

[33] Zheng S., Shen J., Shang Z., Wang W., Xiong Y., Yue X. (2020). Effects of Multi-Step Heat Treatment On the Microstructure, Segregation and Property of a Directional Solidified Ti-45.5Al–3Nb-0.11C-0.3Si Alloy Produced by Electromagnetic Confinement. Mater. Sci. Eng. A.

[34] Mi Y., Wang Y., Wang Y., Dong Y., Chang H., Alexandrov I.V. (2023). Effect of Heat Treatment On Microstructure and Mechanical Behavior of Ultrafine-Grained Ti-2Fe-0.1B. Materials.

[35] Hu M., Wang L., Li G., Huang Q., Liu Y., He J., Wu H., Song M. (2022). Investigations On Microstructure and Properties of Ti-Nb-Zr Medium-Entropy Alloys for Metallic Biomaterials. Intermetallics.

[36] Gierlotka W., Lothongkum G., Lohwongwatana B., Puncreoburt C. (2019). Atomic Mobility in Titanium Grade 5 (Ti6Al4V). J. Min. Metall. Sect. B Metall..

[37] Liu Y., Ge Y., Yu D., Pan T., Zhang L. (2009). Assessment of the diffusional mobilities in bcc Ti–V alloys. J. Alloys Compd..

[38] Shi R., Luo A.A. (2018). Applications of Calphad Modeling and Databases in Advanced Lightweight Metallic Materials. Calphad.

[39] Guo X., Zhou Y., Qi H., Tang X. (2023). Study On the as-Cast Microstructure and Homogenization Mechanism of Gh3128 Superalloy. J. Mater. Eng. Perform..

[40] Liu Y., He S., Li Y., Liu Z., Li C., Li J., Miao H., Zhu D., Su L. (2022). In Vitro Degradation Behavior and Microstructural Evolution of a Novel Biodegradable Zn-Mg-Sr Alloy During Homogenization. J. Mater. Eng. Perform..

[41] Wang T., Wan Z.P., Li Z., Li P.H., Li X.X., Wei K., Zhang Y. (2020). Effect of Heat Treatment Parameters On Microstructure and Hot Workability of as-Cast Fine Grain Ingot of Gh4720Li Alloy. Acta Metall. Sin..

[42] He S., Li C., Ren J., Han Y. (2018). Investigation On Alloying Element Distribution in Cr8Mo2Siv Cold-Work Die Steel Ingot During Homogenization. Steel Res. Int..

